# Rett syndrome (*MECP2*) and succinic semialdehyde dehydrogenase (*ALDH5A1*) deficiency in a developmentally delayed female

**DOI:** 10.1002/mgg3.629

**Published:** 2019-03-04

**Authors:** Madalyn Brown, Paula Ashcraft, Erland Arning, Teodoro Bottiglieri, William McClintock, Frank Giancola, David Lieberman, Natalie S. Hauser, Rebecca Miller, Jean‐Baptiste Roullet, Phillip Pearl, K. Michael Gibson

**Affiliations:** ^1^ Department of Pharmacotherapy, College of Pharmacy and Pharmaceutical Sciences Washington State University Spokane Washington; ^2^ Baylor Scott & White Research Institute Institute of Metabolic Disease Dallas Texas; ^3^ Pediatric Specialists of Virginia Fairfax Virginia; ^4^ Pediatric Care of Northern Virginia Manassas Virginia; ^5^ Department of Neurology Boston Children’s Hospital Harvard School of Medicine Boston Massachusetts; ^6^ Fulgent Genetics Temple City California

**Keywords:** autism spectrum disorder, GABA (γ‐aminobutyric acid), GHB (γ‐hydroxybutyric acid), Rett syndrome, succinic semialdehyde dehydrogenase, succinic semialdehyde dehydrogenase deficiency

## Abstract

**Background:**

We present a patient with Rett syndrome (RTT; *MECP2*) and autosomal‐recessive succinic semialdehyde dehydrogenase deficiency (SSADHD; *ALDH5A1* (aldehyde dehydrogenase 5a1 = SSADH), in whom the current phenotype exhibits features of SSADHD (hypotonia, global developmental delay) and RTT (hand stereotypies, gait anomalies).

**Methods:**

γ‐Hydroxybutyric acid (GHB) was quantified by UPLC‐tandem mass spectrometry, while mutation analysis followed standard methodology of whole‐exome sequencing.

**Results:**

The biochemical hallmark of SSADHD, GHB was increased in the proband's dried bloodspot (DBS; 673 µM; previous SSADHD DBSs (*n* = 7), range 124–4851 µM); control range (*n* = 2,831), 0–78 µM. The proband was compound heterozygous for pathogenic *ALDH5A1* mutations (p.(Asn418IlefsTer39); maternal; p.(Gly409Asp); paternal) and a de novo RTT nonsense mutation in *MECP2* (p.Arg255*).

**Conclusion:**

The major inhibitory neurotransmitter, γ‐aminobutyric acid (GABA), is increased in SSADHD but normal in RTT, although there are likely regional changes in GABA receptor distribution. GABAergic anomalies occur in both disorders, each featuring an autism spectrum phenotype. What effect the SSADHD biochemical anomalies (elevated GABA, GHB) might play in the neurodevelopmental/epileptic phenotype of our patient is currently unknown.

## 1 INTRODUCTION

**Figure 1:**
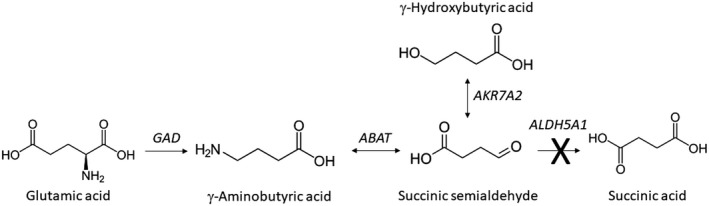
GABA metabolism. Produced from glutamic acid by the action of glutamic acid decarboxylase (*GAD*), γ‐aminobutyric acid (GABA) is metabolized in a two‐step sequence initiated by GABA‐transaminase (*ABAT*; 4‐aminobutyrate aminotransferase) and completed by the action of succinic semialdehyde dehydrogenase (*ALDH5A1*) to generate succinic acid. In patients with deficiency of *ALDH5A1*, succinic semialdehyde is converted to neuroactive γ‐hydroxybutyric acid by the action of aldo‐keto reductase 7a2 (*AKR7A2*)

Succinic semialdehyde dehydrogenase deficiency (SSADHD; *ALDH5A1*; OMIM 271980; HGNC: 408) is a rare, autosomal‐recessive inherited disorder in the GABA pathway (Figure 1; DiBacco et al., 2018).

Prevalent features include hypotonia, developmental delay (especially affecting fine motor skills and expressive language), and typical imaging features involving the globus pallidi. Neuropsychiatric manifestations (OCD, ADHD) are prominent in adolescents and adults. The biochemical hallmarks include elevation of γ‐aminobutyric acid (GABA) and γ‐hydroxybutyric acid (GHB) in physiological fluids and tissues (Novotny, Fulbright, Pearl, Gibson, & Rothman, 2003). The incidence of SSADH deficiency is projected at 1:10^6^, but this may be an underestimate due to undiagnosed patients (Lapalme‐Remis et al., 2015). SSADHD is a static encephalopathy, although an adult subpopulation displays a more severe, progressive course associated with epilepsy (DiBacco et al., 2018).

A neurodevelopmental disorder, Rett syndrome (RTT; OMIM 312750; *MECP2*; HGNC: 6990) encompasses a phenotype of moderate progression associated with severe intellectual and physical disability (Leonard, Cobb, & Downs, 2017). RTT has an X‐linked dominant pattern of inheritance and mainly affects females with an approximate 1:10,000 incidence featuring predominantly de novo mutations in the methyl‐CpG‐binding protein 2 (*MECP2*) gene (Carter et al., 2010; Corchón, Carrillo‐López, & Cauli, 2018; Leonard et al., 2017). Phenotype evolution encompasses initial loss of speech and motor skill regression following apparent normal development, with decelerating head growth, hand stereotypies, gait anomalies (ataxia, apraxia), pulmonary dysfunction, mood fluctuation, and disruptive behavior (Munde, Vlaskamp, & Haar, 2016; Wong, Leonard, Jacoby, Ellaway, & Downs, 2015). We present a patient with SSADHD and RTT, disorders typically associated with autism spectrum disorder and epilepsy (Benke & Möhler, 2018; Frye et al., 2016).

## 2 CASE HISTORY

Parents provided informed consent for this study. The proband was the product of an IVF gestation (BW, 5 lbs. 14.9 oz.; length, 49.5 cm; head circumference 32 cm), Apgars 5/8. There was no birth trauma, although she needed Continuous positive airway pressure (CPAP) briefly, and had low glucose level of 47mg/dl. She was described as vigorous initially, then sleepy at the breast with poor latch. She went home with mother at 48 hr post delivery. She rolled once or twice ~6 months of age but not again, and was not sitting at 9 months of age. At that time, communication skills were delayed. Fine motor skills revealed absence of a pincer grasp and a limited raking grasp to pick up food but then with difficulty successfully transferring to her mouth. She transferred objects from the left to right hand, her dominant one, but not vice‐versa. Her hands were clumsy with intention tremor, intermittent stereotypical finger movements, and internal thumbing, although she had some preserved hand use for playing and feeding. Communication skills encompassed three signs (eat, drink, and all done), and she was able to follow simple commands. She was described as very social, with the ability to read her parents' emotions as well as express her own emotions (e.g., bruxism when stressed). She engaged in social referencing and reciprocal social interactions.

Physical therapy was begun at 9–10 months of age. About that time, and over the next three months, communication declined to the point of losing consonant sounds. She could sit independently at 15 months of age, although shortly thereafter had developmental regression with loss of sounds and much of her limited gross and fine motor skills. At 29 months, she was working on standing independently with a gait trainer and had persistent difficulty with motor planning. The examination at 29 months of age showed acceptable growth with weight at the mean for age and height 15%‐tile, compared to head circumference of 46.5 cm (5%‐tile). There were no dysmorphic or cutaneous stigmata. The motor systems showed decreased axial and appendicular tone with satisfactory strength, limited hand skills, and nondetectable muscle stretch reflexes.

Magnetic resonance imaging of brain at 13 months revealed normal myelination, although there was T2 prolongation and increased signal intensity on the diffusion‐weighted sequence involving the globus pallidus bilaterally. There was slight prominence of the sulci raising suspicion for mild, diffuse parenchymal volume loss. Electroencephalography (EEG) was initially undertaken at 28 months and was interpreted as normal. A follow‐up EEG at 36 months revealed diffuse slowing indicative of encephalopathy with bifrontal sharp slow activity intermittently. Thus far, there have been no seizures. Feeding and occupational therapy have resulted in slow progress in chewing and tongue movements. Hippotherapy, a body suit and a gait trainer have provided assistance with decreased coordination, balance, strength, as well as independent ambulation. An eye gaze computer is enabling some degree of communication/vocalization, and she has shown the capacity to shake her head to indicate “yes”.

## 3 LABORATORY INVESTIGATION

Clinical exome sequencing was initially ordered and performed by Fulgent Diagnostics (Temple City, CA), employing a proprietary method as described (fulgentdiagnostics.com GenBank accession numbers: *ALDH5A1*, NM_001080.3; *MECP2*, NM_004992.3), which confirmed the dual diagnosis at 17 months of age (Table 1). This was ordered in relation to global developmental delay of unknown etiology. Urine organic acids were subsequently requested at 18 months and revealed an elevation of GHB, which was not quantified. GHB was subsequently quantified in patient dried bloodspot (DBS; Forni, Pearl, Gibson, Yu, & Sweetman, 2013). In that study, GHB content in SSADHD DBS was compared with 2,831 archival DBS samples (including newborns, infants and children). Control GHB was 8 ± 5 µM (mean ± *SD*; 99.9%‐tile, 63 µM) (range 0–78 µM), while GHB content in SSADHD DBS was 124–4851 µM (range [*n* = 7]; median 1,182 µM, mean 699 µM). GHB in patient DBS was 673 µM (parallel control, 17 µM), 10‐fold above the 63 µM cutoff.

**Table 1:** Whole‐exome sequence analysis of proband and parents

Family member	Gene	Variant	Zygosity and inheritance	Comment or Reference
Proband	*ALDH5A1*	NM_001080.3:g.1253del p.(Asn418IlefsTer39)	Hz, AR	Protein termination
	*ALDH5A1*	NM_001080.3;c.1226G>A p.(Gly409Asp)	Hz, AR	Hogema et al. (2001) (<1% residual *ALDH5A1* activity)
	*MECP2*	NM_004992.3;c.763C>T p.Arg255*	Hz, XL	Christodoulou & Ho (2012) Protein termination
Mother	*ALDH5A1*	NM_001080.3:g.1253del p.(Asn418IlefsTer39)	Hz, AR	Protein termination
	*MECP2*	NM_004992.3;c.763C>T p.Arg255*	Hz, XL	Not detected
Father	*ALDH5A1*	NM_001080.3;c.1226G>A p.(Gly409Asp)	Hz, AR	Hogema et al. (2001) (<1% residual *ALDH5A1* activity)
	*MECP2*	NM_004992.3;c.763C>T p.Arg255*	Hz, XL	Not detected

Note

Hz, heterozygosity; AR, autosomal recessive; XL, X‐linked; all variants are predicted to be pathogenic. GenBank accession numbers: *ALDH5A1*, NM_001080.3; *MECP2*, NM_004992.3

## 4 DISCUSSION

During the first 3.5 years of development, the phenotype of our patient appears similar to the sum of the two disorders rather than synergistically worse. Her loss of hand function, loss of speech, regression in ambulation and hand stereotypies clearly meet diagnostic criteria for classic RTT. Hypotonia, dysphagia, kyphosis, and sleep difficulties which are noted in our patient and are frequently seen in RTT (Carter et al., 2010; Neul et al., 2010). Many of the unique features of RTT, e.g., breathing abnormalities, are not present, although she does exhibit bruxism, is variably challenged with apraxia, and demonstrates occasional intention tremor when using her hands. Of interest, she does not exhibit any of the characteristic social delays often present in autism spectrum disorder, RTT, or SSADHD. The patient's mother describes her as having a strong sense of social reciprocity, initiation and maintenance of social routines, and with good nonverbal communication (considering her physical limitations) such as eye contact. She can understand emotion on the faces of others and responds appropriately to changes in affect with other individuals.

RTT and SSADHD likely share imbalanced excitatory versus inhibitory neurotransmission (Benke & Möhler, 2018; Frye et al., 2016), perhaps underpinning to some degree the autism spectrum and epilepsy phenotype of both disorders. Of interest, patients with SSADHD have downregulated GABA(A) and GABA(B) receptors (Pearl et al., 2009; Reis et al., 2012). Similarly, RTT patients demonstrate lowered expression of GABA(A) receptors (Blue, Naidu, & Johnston, 1999; Yamashita et al., 1998), and transcranial magnetic stimulation studies suggest abnormal GABA(B) receptor function (Krajnc & Zidar, 2016). In the *Mecp2*‐null mouse, there is evidence for fewer synaptic GABA(A) receptors (Jin, Cui, Zhong, Jin, & Jiang, 2013) and more extrasynaptic receptors, at least in the nucleus tractus solitarius (Chen et al., 2018), with upregulated response of postsynaptic GABA(A) receptors in the barrel cortex of *Mecp2*‐null mice (Lo et al., 2016). There is also a reported downregulation of synaptic GABA transporters in *Mecp2*‐null mice (Kang et al. 2014). Biochemically, patients with SSADHD have increased GABA (Novotny et al., 2003), while GABA levels in CSF from RTT patients are normal (Perry, Dunn, Ho, & Crichton, 1988). The effect of increased GABA on the RTT neurodevelopmental phenotype (exacerbation, attenuation) remains to be determined in our patient.

## CONFLICT OF INTEREST

Madalyn Brown, Paula Ashcraft, Erland Arning, Teodoro Bottiglieri, William McClintock, Frank Giancola, David Lieberman, Natalie Hauser, Rebecca Miller, Jean‐Baptiste Roullet, Phillip Pearl, and K. Michael Gibson declare that they have nothing to disclose and no conflict of interest.

## References

[mgg3629-bib-0001] Benke, D. , & Möhler, H. (2018). Impact on GABA systems in monogenetic developmental CNS disorders: Clues to symptomatic treatment. Neuropharmacology, 136(Pt A), 46–55. 10.1016/j.neuropharm.2017.07.030 28764992

[mgg3629-bib-0002] Blue, M. E. , Naidu, S. , & Johnston, M. V. (1999). Altered development of glutamate and GABA receptors in the basal ganglia of girls with Rett syndrome. Experimental Neurology, 156, 345–352. 10.1006/exnr.1999.7030 10328941

[mgg3629-bib-0003] Carter, P. , Downs, J. , Bebbington, A. , Williams, S. , Jacoby, P. , Kaufmann, W. E. , & Leonard, H. (2010). Stereotypical hand movements in 144 subjects with Rett syndrome from the population‐based Australian database. Movement Disorders, 25, 282–288. 10.1002/mds.22851 19908321

[mgg3629-bib-0004] Chen, C. Y. , Di Lucente, J. , Lin, Y. C. , Lien, C. C. , Rogawski, M. A. , Maezawa, I. , & Jin, L. W. (2018). Defective GABAergic neurotransmission in the nucleus tractus solitarius in Mecp2-null mice, a model of Rett syndrome. Neurobiology of Disease, 109, 25–32. 28927958 10.1016/j.nbd.2017.09.006 PMC5696074

[mgg3629-bib-0005] Christodoulou, J. , & Ho, G. (2012). MECP2‐related disorders In Adam M. P., Ardinger H. H., Pagon R. A., Wallace S. E., Bean L. J. H., Stephens K., & Amemiya A. (Eds.), GeneReviews® [Internet]. Seattle, WA: University of Washington, Seattle; 1993–2018. 2001 Oct 3 [updated 2012 Jun 28].

[mgg3629-bib-0006] Corchón, S. , Carrillo‐López, I. , & Cauli, O. (2018). Quality of life related to clinical features in patients with Rett syndrome and their parents: A systematic review. Metabolic Brain Disease, 33, 1801–1810. 10.1007/s11011-018-0316-1 30220073

[mgg3629-bib-0007] DiBacco, M. L. , Roullet, J.‐B. , Kapur, K. , Brown, M. N. , Walters, D. C. , Gibson, K. M. , & Pearl, P. L. (2018). Age‐related phenotype and biomarker changes in SSADH deficiency. Annals of Clinical and Translational Neurology, 6, 114–120. 10.1002/acn3.696 30656189 PMC6331944

[mgg3629-bib-0008] Forni, S. , Pearl, P. L. , Gibson, K. M. , Yu, Y. , & Sweetman, L. (2013). Quantitation of gamma‐hydroxybutyric acid in dried blood spots: Feasibility assessment for newborn screening of succinic semialdehyde dehydrogenase (SSADH) deficiency. Molecular Genetics and Metabolism, 109, 255–259. 10.1016/j.ymgme.2013.05.002 23742746 PMC3881544

[mgg3629-bib-0009] Frye, R. E. , Casanova, M. F. , Fatemi, S. H. , Folsom, T. D. , Reutiman, T. J. , Brown, G. L. , … Adams, J. B. (2016). Neuropathological mechanisms of seizures in autism spectrum disorder. Frontiers in Neuroscience, 10, 192 10.3389/fnins.2016.00192 27242398 PMC4861974

[mgg3629-bib-0010] Hogema, B. M. , Gupta, M. , Senephansiri, H. , Burlingame, T. G. , Taylor, M. , Jakobs, C. , … Gibson, K. M . (2001). Pharmacologic rescue of lethal seizures in mice deficient in succinate semialdehyde dehydrogenase. Nature Genetics, 29(2), 212–216. 11544478 10.1038/ng727

[mgg3629-bib-0011] Jin, X. , Cui, N. , Zhong, W. , Jin, X. T. , & Jiang, C. (2013). GABAergic synaptic inputs of locus coeruleus neurons in wild‐type and Mecp2‐null mice. American Journal of Physiology. Cell Physiology, 304, C844–C857. 23392116 10.1152/ajpcell.00399.2012 PMC3651605

[mgg3629-bib-0012] Kang, S. K. , Kim, S. T. , Johnston, M. V. , & Kadam, S. D. (2014). Temporal- and Location-Specific Alterations of the GABA Recycling System in Mecp2 KO Mouse Brains. Journal of Central Nervous System Disease, 6, 21–28. 24737935 10.4137/JCNSD.S14012 PMC3981570

[mgg3629-bib-0013] Krajnc, N. , & Zidar, J. (2016). The role of transcranial magnetic stimulation in evaluation of motor cortex excitability in Rett syndrome. European Journal of Paediatric Neurology, 20, 597–603. 10.1016/j.ejpn.2016.03.010 27131828

[mgg3629-bib-0014] Lapalme‐Remis, S. , Lewis, E. C. , De Meulemeester, C. , Chakraborty, P. , Gibson, K. M. , Torres, C. , … Pearl, P. L. (2015). Natural history of succinic semialdehyde dehydrogenase deficiency through adulthood. Neurology, 85, 861–865. 10.1212/WNL.0000000000001906 26268900 PMC4560056

[mgg3629-bib-0015] Leonard, H. , Cobb, S. , & Downs, J. (2017). Clinical and biological progress over 50 years in Rett syndrome. Nature Reviews. Neurology, 13, 37–51. 10.1038/nrneurol.2016.186 27934853

[mgg3629-bib-0016] Lo, F. S. , Blue, M. E. , & Erzurumlu, R. S. (2016). Enhancement of postsynaptic GABAA and extrasynaptic NMDA receptormediated responses in the barrel cortex of Mecp2-null mice. Journal of Neurophysiology, 115(3), 1298–1306. 26683074 10.1152/jn.00944.2015 PMC4808090

[mgg3629-bib-0017] Munde, V. , Vlaskamp, C. , & Ter Haar, A. (2016). Social‐emotional instability in individuals with Rett syndrome: Parents' experiences with second stage behaviour. Journal of Intellectual Disability Research, 60, 43–53. 10.1111/jir.12233 26497300

[mgg3629-bib-0018] Neul, J. L. , Kaufmann, W. E. , Glaze, D. G. , Christodoulou, J. , Clarke, A. J. , Bahi‐Buisson, N. , … Percy, A. K. (2010). Rett syndrome: Revised diagnostic criteria and nomenclature. Annals of Neurology, 68, 944–950. 10.1002/ana.22124 21154482 PMC3058521

[mgg3629-bib-0019] Novotny, E. J. Jr , Fulbright, R. K. , Pearl, P. L. , Gibson, K. M. , & Rothman, D. L. (2003). Magnetic resonance spectroscopy of neurotransmitters in human brain. Annals of Neurology, 54(Suppl 6), S25–31. 12891651 10.1002/ana.10697

[mgg3629-bib-0020] Pearl, P. L. , Gibson, K. M. , Quezado, Z. , Dustin, I. , Taylor, J. , Trzcinski, S. , … Theodore, W. (2009). Decreased GABA‐A binding on FMZ‐PET in succinic semialdehyde dehydrogenase deficiency. Neurology, 73, 423–429. 10.1212/WNL.0b013e3181b163a5 19667317 PMC2727143

[mgg3629-bib-0021] Perry, T. L. , Dunn, H. G. , Ho, H. H. , & Crichton, J. U. (1988). Cerebrospinal fluid values for monoamine metabolites, gamma‐aminobutyric acid, and other amino compounds in Rett syndrome. Journal of Pediatrics, 112, 234–238. 2448442 10.1016/s0022-3476(88)80060-x

[mgg3629-bib-0022] Reis, J. , Cohen, L. G. , Pearl, P. L. , Fritsch, B. , Jung, N. H. , Dustin, I. , & Theodore, W. H. (2012). GABAB‐ergic motor cortex dysfunction in SSADH deficiency. Neurology, 79, 47–54. 10.1212/WNL.0b013e31825dcf71 22722631 PMC3385496

[mgg3629-bib-0023] Wong, K. , Leonard, H. , Jacoby, P. , Ellaway, C. , & Downs, J. (2015). The trajectories of sleep disturbances in Rett syndrome. Journal of Sleep Research, 24, 223–233. 10.1111/jsr.12240 25219940 PMC4351186

[mgg3629-bib-0024] Yamashita, Y. , Matsuishi, T. , Ishibashi, M. , Kimura, A. , Onishi, Y. , Yonekura, Y. , & Kato, H. (1998). Decrease in benzodiazepine receptor binding in the brains of adult patients with Rett syndrome. Journal of the Neurological Sciences, 154, 146–150. 10.1016/S0022-510X(97)00223-2 9562304

